# NTRU-MCF: A Chaos-Enhanced Multidimensional Lattice Signature Scheme for Post-Quantum Cryptography

**DOI:** 10.3390/s25113423

**Published:** 2025-05-29

**Authors:** Rong Wang, Bo Yuan, Minfu Yuan, Yin Li

**Affiliations:** 1 Guangzhou Institute of Software, Guangzhou 510006, China; wangrong@gzis.ac.cn (R.W.); yuanminfu@gzis.ac.cn (M.Y.); 2 School of Computer Science and Engineering, Beihang University, Beijing 100191, China; boyuan@buaa.edu.cn; 3 School of Software Engineer, South China University of Technology, Guangzhou 511458, China; 4 Guangzhou Zhongke Yide Technology Co., Ltd., Guangzhou 511458, China

**Keywords:** post-quantum cryptography, multidimensional lattices, fractional-order chaos, NTRU-MCF, signature scheme, quantum resistance

## Abstract

To address the growing threat of quantum computing to classical cryptographic primitives, this study introduces NTRU-MCF, a novel lattice-based signature scheme that integrates multidimensional lattice structures with fractional-order chaotic systems. By extending the NTRU framework to multidimensional polynomial rings, NTRU-MCF exponentially expands the private key search space, achieving a key space size $\geq 2^{256}$ for dimensions m≥2 and rendering brute-force attacks infeasible. By incorporating fractional-order chaotic masks generated via a hyperchaotic Lü system, the scheme introduces nonlinear randomness and robust resistance to physical attacks. Fractional-order chaotic masks, generated via a hyperchaotic Lü system validated through NIST SP 800-22 randomness tests, replace conventional pseudorandom number generators (PRNGs). The sensitivity to initial conditions ensures cryptographic unpredictability, while the use of a fractional-order L hyperchaotic system—instead of conventional pseudorandom number generators (PRNGs)—leverages multiple Lyapunov exponents and initial value sensitivity to embed physically unclonable properties into key generation, effectively mitigating side-channel analysis. Theoretical analysis shows that NTRU-MCF’s security reduces to the Ring Learning with Errors (RLWE) problem, offering superior quantum resistance compared to existing NTRU variants. While its computational and storage complexity suits high-security applications like military and financial systems, it is less suitable for resource-constrained devices. NTRU-MCF provides robust quantum resistance and side-channel defense, advancing PQC for classical computing environments.

## 1. Introduction

The rapid advancement of quantum computing poses a critical threat to classical public-key cryptosystems such as RSA and ECC. Shor’s algorithm [1], which solves integer factorization and discrete logarithm problems in polynomial time, has propelled post-quantum cryptography (PQC) to the forefront of cryptographic research. Among PQC candidates, lattice-based cryptography stands out due to its robustness against quantum attacks, relying on hard problems such as the Shortest Vector Problem (SVP) and Learning with Errors (LWE) [2]. Since Ajtai’s seminal work [3] established the link between lattice cryptography and worst-case complexity theory, lattice-based signature schemes have evolved through two primary paradigms. In the Hash-and-Sign paradigm, the GPV framework [4] introduced trapdoor preimage sampling for constructions based on the Small Integer Solution (SIS) problem. Subsequent optimizations include Alwen and Peikert’s Gaussian sampling [5], Micciancio and Peikert’s matrix trapdoor generation algorithm (MP12) [6], and the Ducas–Lyubashevsky–Prest scheme [7], which reduced trapdoor size by 40% through ring algebraic structures and improved signature generation efficiency to $10^{3}$ signatures per second [8].

Post-quantum cryptography (PQC) encompasses classical cryptographic schemes designed to resist attacks from both classical and quantum computers. Unlike quantum cryptography, which leverages quantum mechanical principles (e.g., quantum key distribution), PQC relies on mathematical problems—such as those in lattice-based cryptography—that are conjectured to be intractable even for quantum algorithms. Lattice-based schemes, including NTRU-MCF, operate entirely on classical computers, using problems like the Shortest Vector Problem (SVP) and Ring Learning with Errors (RLWE) to achieve security against quantum threats, such as Shor’s algorithm for factoring and discrete logarithms. This classical foundation ensures compatibility with existing computing infrastructure while addressing the growing threat of quantum computing. In the Fiat-Shamir paradigm, Lyubashevsky’s trapdoor-free construction [9] eliminated precomputation overhead but required rejection sampling. Notably, standardized schemes like Dilithium [10] and Falcon [11] have demonstrated superior performance in NIST evaluations. For instance, Falcon achieves public key sizes <1 KB and signature lengths <0.5 KB on general computing platforms through specialized NTRU lattice constructions [12]. Despite these advancements, existing lattice-based schemes remain vulnerable to quantum-assisted SVP solvers such as the Kannan-Helfrich algorithm [13] and face challenges in providing integrated defenses against physical attacks like side-channel analysis. Recent studies [14,15] indicate that increasing lattice dimensions exponentially amplifies SVP complexity, offering a promising avenue for enhanced quantum resistance.

However, current schemes often sacrifice computational efficiency and lack integrated designs that balance quantum resistance with robust physical attack defense. Furthermore, while the proliferation of IoT and real-time systems motivates the need for advanced cryptographic solutions, we note that the complexity and storage overhead of multidimensional lattice schemes such as NTRU-MCF are fundamentally unsuitable for severely resource-constrained or real-time embedded devices. Instead, our approach aims at high-assurance environments, such as critical infrastructure and defense, where security requirements outweigh resource limitations. In contrast, standardized schemes like Falcon and Dilithium, which offer compact key and signature sizes and high efficiency, remain preferable for mass-market and IoT deployment.

To address these limitations, this paper proposes NTRU-MCF, a novel post-quantum signature scheme combining multidimensional lattice structures and fractional-order chaotic systems, tailored for scenarios prioritizing maximum security over resource efficiency. By extending NTRU lattices to multidimensional polynomial rings, NTRU-MCF exponentially increases the private key search space, making brute-force attacks computationally infeasible and significantly elevating the complexity of lattice-based attacks, offering an estimated quantum resistance exceeding $2^{256}$ for dimensions m≥2. This multidimensional extension directly enhances resistance to quantum SVP algorithms, where complexity grows superlinearly with dimension. Simultaneously, the integration of fractional-order chaotic systems, specifically leveraging the non-periodic dynamics and extreme sensitivity to initial conditions of hyperchaotic Lü systems, introduces strong nonlinear randomness. The cryptographic randomness of these chaotic sequences is validated through NIST SP 800-22 tests, ensuring their suitability for secure key generation and masking.

The main contributions of this paper are summarized as follows.

- **Multidimensional lattice extension**: The one-dimensional NTRU lattice is extended to multidimensional polynomial rings, $R=Z\left[x_{1},...,x_{m}\right]/\left(x_{1}^{N_{1}}-1,...,x_{m}^{N_{m}}-1\right)$, leveraging the exponentially increasing complexity of solving SVP in high-dimensional lattices (m≥2) to provide enhanced resistance against both classical and quantum attacks. For m≥2, the key space size is theoretically $\geq 2^{256}$.
- **Chaos-enhanced randomness and side-channel security**: Fractional-order logistic maps and Lü hyperchaotic systems are integrated into key generation and encryption mask generation. Their non-periodic dynamics and extreme initial condition sensitivity generate highly unpredictable sparse polynomial coefficients and masks, effectively replacing PRNGs to eliminate deterministic patterns and providing a strong defense against side-channel analysis.
- **Comprehensive security and performance analysis**: The scheme’s security is formally shown to be reducible to the RLWE problem, demonstrating superior quantum resistance, an expanded key space, and enhanced side-channel defense compared to existing NTRU variants. Performance and storage efficiency are analyzed through theoretical complexity bounds, highlighting the trade-offs inherent in achieving higher security levels.

The remainder of this paper is organized as follows: Section 2 reviews related work; Section 3 formalizes the security model and assumptions; Section 4 details the multidimensional lattice construction, chaotic mask generation, and protocol workflow of NTRU-MCF; Section 5 provides security proofs based on lattice reduction attacks and quantum complexity theory; Section 6 evaluates performance and storage efficiency through theoretical analysis; and Section 7 concludes the paper and outlines future research directions.

## 2. Related Works

The NTRU cryptosystem, proposed by Hoffstein et al. in 1996 [12], relies on the SVP and Closest Vector Problem (CVP) in lattices. Early NTRU-based signature schemes, such as NSS [16], faced vulnerabilities to statistical attacks [17] and signature forgery [18]. Improved variants like NTRUSign (2003) [19] achieved secure signatures through parameter optimization but remained constrained by key size and computational efficiency [20]. The GPV framework [21] pioneered trapdoor sampling techniques for lattice signatures [22]. Subsequent advancements include Alwen and Peikert’s Gaussian sampling optimizations [23], MP12’s small random matrices [24], and Chen et al.’s approximate trapdoor generation algorithm [25], which reduced trapdoor dimensions by  50% under equivalent security levels [26]. The current leading schemes, Dilithium [10] and Falcon [11], prioritize practicality: Falcon generates >1000 signatures per second on standard hardware with compact keys. While highly efficient, these schemes primarily focus on provable security against mathematical attacks and often lack explicit, integrated mechanisms to counteract physical attacks such as side-channel analysis, which are particularly relevant in hardware implementations and embedded systems. This gap motivates the need for schemes like NTRU-MCF that build in side-channel resistance from the ground up.

Recent research has expanded NTRU’s utility in homomorphic encryption [27], blind signatures [28], and linkable ring signatures (LRSs). For example, Ye et al. [29] reduced LRS signature length from O(n log q) to O(n) while improving verification efficiency by 40%. In 2024, Kim et al. [30] combined NTRU-LRS with verifiable random functions (VRFs) for hybrid blockchain authentication, showcasing NTRU’s cross-domain potential. Advancements in trapdoor generation have further optimized NTRU signatures. Ducas et al. [31] combined NTRU lattice trapdoors with fast Fourier orthogonalization [32], tripling Gaussian sampling efficiency. Integration with secure multi-party computation (MPC) and blockchain technologies has also emerged, with Jiang et al. [33] developing an NTRU-MKFHE-based MPC protocol for blockchain, while Parthasarathy et al. [34] implemented a medical data-sharing system using NTRU signatures and MPC.

The application of chaotic systems in cryptography is a well-explored area, often leveraging their properties of sensitivity to initial conditions, unpredictability, and complex dynamics for tasks like pseudorandom number generation, image encryption, and secure communication. For instance, Yu et al. [35] explores a PRNG based on hyperchaotic systems. However, integrating chaotic systems directly into lattice-based schemes, particularly in the context of post-quantum signatures and side-channel resistance, remains a less explored domain. Existing chaotic cryptosystems often face challenges related to finite precision effects, small key space issues, and the lack of rigorous security proofs against dedicated cryptanalytic attacks. Our work differs by specifically employing fractional-order chaotic systems to enhance the randomness and unpredictability of critical components within a well-established lattice framework (NTRU), aiming to build resistance against both quantum and physical attacks without compromising the underlying lattice-based security guarantees. The use of fractional-order systems, with their potentially richer dynamics and higher complexity compared to integer-order systems, offers a distinct approach to generating the required cryptographic randomness for lattice-based constructions.

## 3. Safety Model

### 3.1. Safety Assumptions

The security of the proposed scheme relies on two foundational assumptions: (1) the computational intractability of problems in multidimensional lattices and (2) the unpredictability of fractional-order chaotic systems.

#### 3.1.1. Computational Hardness in Multidimensional Lattices

A multidimensional polynomial ring framework $R=Z\left[x_{1},...,x_{m}\right]/\left(x_{1}^{N_{1}}-1,...,x_{m}^{N_{m}}-1\right)$ significantly amplifies computational complexity compared to traditional one-dimensional counterparts (e.g., NTRU). The Approximate Shortest Vector Problem (Approx-SVP), which involves finding a vector *v* in lattice L(R) with length $\left|v\right|\leq \gamma \cdot \lambda_{1}\left(L\right)$ (where γ is a polynomial multiple), is a classic hard problem in cryptography. Specifically, attackers need to search for vectors meeting certain conditions in high-dimensional spaces, with the search complexity growing exponentially with the lattice dimension *m*. For multidimensional lattices L(R) with dimension m≥3 and modulus q=2048, there are no known polynomial-time algorithms (including quantum algorithms) that can find vectors with length $∥v∥\leq \gamma \cdot \lambda_{1}\left(L\right)$ with probability ϵ>negl(λ) (where γ=poly(n), and λ is the security parameter). Existing research indicates that the complexity of Approx-SVP for high-dimensional tensor product lattices has a superlinear relationship with dimension *m*. When m=3 and $N_{i}=256$, the quantum algorithm complexity lower bound is $\Omega (2^{0.35\cdot m\cdot N})$. As there are currently no effective quantum algorithms capable of solving the Approx-SVP problem in polynomial time, signature schemes based on multidimensional lattices theoretically offer resistance to quantum attacks.

#### 3.1.2. Unpredictability of Fractional-Order Chaotic Systems

The fractional-order Lü-system-generated sequences are assumed to satisfy the following properties:

1. **Initial value sensitivity**: A minor deviation ($\delta >10^{-6}$) in the initial seed induces statistical independence in subsequent outputs. For the fractional-order Lü system, a slight change in the initial seed (e.g., $\delta >10^{-6}$) will cause the sequence to become statistically independent after 𝒪(log(1/δ)) steps. Even if an attacker obtains some sequence information, it will be difficult for them to deduce the initial seed.
2. **Chaotic sequence randomness**: Sequence generation depends on control parameters (e.g., $a_{i},b_{i},c_{i},q_{i}$) and initial seeds. The nonlinear dynamics of fractional-order systems preclude parameter or seed reconstruction from limited outputs. Given a discretization coefficient ${coef}_{i,j}\in \left\{-1,0,1\right\}$, recovering $a_{i},b_{i},c_{i},q_{i}$ or $x_{0}$ with non-negligible probability is computationally infeasible.

### 3.2. Security Analysis

Multidimensional lattice-based cryptography extends the NTRU protocol into higher-dimensional spaces, enhancing quantum resistance and security robustness. The framework comprises five core processes: key generation, encryption, decryption, signature generation, and verification, all underpinned by the hardness of lattice problems.

**(1) Key generation**

Parameter selection: polynomial degree, *N*, moduli, *q* (large) and *p* (small), and basis vectors, $b_{1},b_{1},...,b_{d}$, in *d*-dimensional space.

Key derivation: the public key $h\left(x\right)$ is computed as $h\left(x\right)=p\cdot g\left(x\right)\cdot f^{-1}\left(x\right)\;\mathrm{mod}\;q$, where $f\left(x\right)$ (private key) and $g\left(x\right)$ (random polynomial) are small-norm polynomials.

**(2) Encryption**

A message, m(x), is encoded as a multidimensional vector. Encryption involves selecting a random polynomial, r(x), (bounded by *p*), and computing the following:

(1) e(x)=r(x)·h(x)+m(x)modq

where h(x) is a public parameter, r(x) is a random polynomial selected from a small modulus, p, and the ciphertext e(x) consists of two parts: one part is the product of the random polynomial and the public key, and the other part is the message polynomial.

**(3) Decryption**

During the decryption process, the private key polynomial f(x) is used to recover the message. Under the assumption that the received ciphertext is e(x), the private key f(x) is used to calculate the intermediate value.

(2) a(x)=e(x)·f(x)modq

Then, a(x) is divided by *p*, and the modulus is taken to obtain the following:

(3) b(x)=a(x)modq

Finally, the plaintext message is recovered.

(4) $$m\left(x\right)=f_{p}^{-1}\left(x\right)\cdot b\left(x\right)\;\mathrm{mod}\;p$$

In this context, $f_{p}^{-1}\left(x\right)$ is the inverse of the private key polynomial f(x) modulo *p*.

**(4) Signature generation**

For message m(x), compute its hash H(m(x)), and then generate the signature:

(5) s(x)=H(m(x))·f(x)modq

**(5) Signature verification**

Use the public key to verify the signature, and determine whether v(x) is equal to H(m(x))·f(x)modq. If they are equal, the signature is valid. The formula is as follows:

(6) v(x)=s(x)·h(x)modq

(7) $$v\left(x\right)\overset{?}{=}H\left(m\left(x\right)\right)\cdot f\left(x\right)\;\left(\mathrm{mod}\;q\right)$$

**(6) Quantum resistance**

Security hinges on the hardness of lattice problems (SVP/CVP), which remain intractable even for quantum computers. Thus, compared to number-theoretic cryptosystems (e.g., RSA and ECC), NTRU-based schemes offer superior post-quantum security.

### 3.3. Confidentiality Analysis

#### 3.3.1. Public–Private Key Relationship Complexity

The relationship between the public key h and the private key f is as follows:

(8) h=pf+gmodq

The solution of the inverse element of the private key f depends on the extended Euclidean algorithm, which has high computational complexity in the multidimensional polynomial ring. Attackers cannot effectively derive the private key f only through the public key h, thus ensuring the confidentiality of the private key.

#### 3.3.2. Chaotic System Randomness

The private key f is generated via a fractional order chaotic system, and the chaotic sequence is defined via the following formula:

(9) $$x_{n+1}=x_{n}\cdot e^{\alpha (1-x_{n})}\cdot \Gamma \left(1-q\right)\sum_{k=0}^{n}\frac{{\left(-1\right)}^{k}\Gamma \left(q+1\right)}{k!\Gamma (q-k+1)}x_{n-k}$$

The output of a chaotic system is non-periodic and sensitive to initial values. Even if an attacker obtains part of the output sequence, they still cannot predict the values of other sequences, ensuring the randomness and unpredictability of the key.

### 3.4. Unforgeability Analysis

#### 3.4.1. Signature Security

The signature s is calculated through the following formula:

(10) s=H(m)·fmodq

Since H(m) is the unique hash value of the message, and f is an unpredictable private key, an attacker cannot forge a legitimate signature. If an attacker attempts to forge a signature, $s^{\prime }$, they must meet the following conditions:

(11) $$s^{\prime }\equiv H\left(m\right)\cdot p\;\mathrm{mod}\;q$$

However, in the case where the attacker does not know f, forging a signature is equivalent to solving the discrete logarithm problem or the SVP problem, which is not feasible under the existing computational model.

#### 3.4.2. Verification Reliability

The process of verifying a signature is as follows:

(12) v=s·hmodq

The verifier checks whether the following equation is valid:

(13) v≡H(m)·pmodq

Any attempt to tamper with the signature s or the message m will result in a failed verification, thereby ensuring the non-forgeability of the signature.

### 3.5. Anonymity Analysis

#### 3.5.1. Identity Obfuscation

Signatures depend solely on the private key f and the message m hash H(m), with no identity-linked data in the signature itself. Attackers cannot infer the identity of the signatory by observing the signature. The initial value sensitivity and randomness of the fractional-order chaotic system further conceal the generation process of the signature, enhancing its anonymity.

#### 3.5.2. Resistance to Linkage Attacks

High-dimensional chaotic seeds ensure distinct trajectories for different signers. Even with multiple signatures, adversaries cannot correlate them to a single entity due to the non-periodic and unpredictable nature of the chaotic sequences used in key generation.

### 3.6. Linkability Analysis

#### 3.6.1. Signature Linkability

Identical messages signed by the same private key share a mathematical relationship through the hash function H(m), enabling deterministic linkage. This property is crucial for applications requiring signature linkability, such as in some blockchain implementations.

#### 3.6.2. Replay Attack Prevention

Since the signature s contains the message hash value H(m), the signatures of different messages are independent of each other. Attackers cannot forge new messages using old signatures s, and the uniqueness and independence of signatures ensure the ineffectiveness of replay attacks. The scheme ensures that each signature is bound to its specific message, preventing unauthorized reuse.

## 4. Lattice-Based Cryptographic Signature Scheme with NTRU-MCF

The NTRU-MCF scheme, a lattice-based post-quantum cryptographic protocol, comprises three core algorithms detailed in Algorithms 1–3: key generation (Algorithm 1), encryption/decryption (Algorithm 2), and signature/verification (Algorithm 3). These algorithms form the complete framework for NTRU-MCF’s cryptographic operations, leveraging multidimensional lattice structures and fractional-order chaotic systems to achieve security reducible to the Ring Learning with Errors (RLWE) problem. Designed to run on classical computers, these algorithms are compatible with standard hardware and software environments, ensuring practical deployment. Their security against quantum attacks stems from the intractability of lattice problems like the shortest vector problem (SVP), which remain hard even for quantum algorithms, providing robust protection against quantum computing threats.

### 4.1. Extended Multidimensional Lattice Structure

Traditional NTRU employs a one-dimensional convolution polynomial ring. To enhance security, we extend this structure to a multidimensional lattice, where keys and message polynomials reside in a multivariate polynomial ring. Under this framework, key generation, encryption, and decryption operations are defined as follows:

(14) $$R=Z\left[x_{1},x_{2},\ldots ,x_{m}\right]/\left(x_{1}^{N_{1}}-1,\ldots ,x_{m}^{N_{m}}-1\right)$$

Let $x_{1},x_{2},\ldots ,x_{m}$ denote independent variables, where m>1 increases lattice complexity and resistance to quantum attacks. The private key $f(x_{1},\ldots ,x_{m})$ and public key $h(x_{1},\ldots ,x_{m})$ in this structure are generated as follows:

(15) $$h\left(x_{1},\ldots ,x_{m}\right)=g\left(x_{1},\ldots ,x_{m}\right)\cdot f^{-1}\left(x_{1},\ldots ,x_{m}\right)\;\mathrm{mod}\;q$$

### 4.2. Fractional-Order Chaotic Systems

Traditional NTRU relies on pseudo-random number generators (PRNGs) for key generation, which exhibit periodicity and predictability risks. To address this limitation, we propose the integration of fractional-order chaotic systems. These systems leverage nonlinear dynamics, aperiodicity, and extreme sensitivity to initial conditions, thereby enhancing cryptographic security.

#### 4.2.1. Fractional-Order Logistic Map

The fractional-order Logistic map is a fractional-order extension of the classic Logistic map, which is expressed as follows:

(16) $$x_{n+1}^{\left(i\right)}=x_{n}^{\left(i\right)}\cdot e^{\alpha_{i}\left(1-x_{n}^{\left(i\right)}\right)}$$

where $x_{n}^{\left(i\right)}$ is the state variable of the system, and $\alpha_{i}$ is the control parameter that determines the chaotic behavior of the system.

Among them, $x_{n}^{\left(i\right)}$ is the state variable of the system, and $\alpha_{i}$ is the control parameter that determines the chaotic behavior of the system. $e^{\alpha_{i}\left(1-x_{n}^{\left(i\right)}\right)}$ is an exponentially nonlinear term, which takes the form of an exponential function. As the parameter $\alpha_{i}$ changes, the system will experience a transition from stable periodicity to chaos. Chaotic behavior is relatively simple, usually manifested as a single-peak mapping.

We propose using a discrete chaotic mapping described by a fractional difference equation, and the key generation formula is as follows:

(17) $$x_{n+1}=x_{n}\cdot e^{\alpha (1-x_{n})}\cdot \Gamma \left(1-q\right)\sum_{k=0}^{n}\frac{{\left(-1\right)}^{k}\Gamma \left(q+1\right)}{k!\Gamma (q-k+1)}x_{n-k}$$

where α∈(0,4]) is the chaos control parameter; when α>3.2, the system enters the chaotic state. q∈(0,1) is the order of the fractional order, and reducing q can enhance nonlinearity. Γ(·) is the Gamma function, which is used for fractional order difference calculation. The sequence xn takes values in the range of (0,1), and it has the properties of ergodicity and initial value sensitivity.

#### 4.2.2. Fractional-Order Lü Hyperchaotic System

The fractional-order Lü hyperchaotic system is defined as a three-dimensional continuous system:

(18) $$\left\{\begin{array}{c} D^{q}x=a\left(y-x\right)+z\\ D^{q}y=cy-xz\\ D^{q}z=xy-bz \end{array}\right.$$

where $D^{q}$ denotes the Caputo fractional derivative, a,b,c are system parameters, and *q* is the fractional order, and q∈(0,1) (e.g., q=0.9). The fractional-order Lü hyperchaotic system exhibits enhanced chaotic complexity characterized by hyperchaotic phenomena, manifested through the coexistence of multiple positive Lyapunov exponents. Its chaotic attractor demonstrates intricate topological features, typically manifesting as high-dimensional surfaces or fractal structures. The incorporation of fractional calculus through order parameter q amplifies both the system’s nonlinear complexity and trajectory unpredictability.

#### 4.2.3. Randomness Validation and Finite Precision of Fractional-Order Chaotic Systems

To ensure the cryptographic suitability of the fractional-order Lü hyperchaotic system as a replacement for traditional PRNGs, we conducted statistical randomness tests using the NIST SP 800-22 test suite. A sequence of $10^{6}$ bits was generated from the discretized output of the Lü system with parameters a=36, b=3, c=20, and q=0.9 and initial conditions derived from a SHA-3 seed. We validated their output sequences using the NIST SP 800-22 statistical test suite. The test suite was applied to sequences generated via the fractional-order Lü system with double-precision floating-point arithmetic, typical of cryptographic implementations. All relevant tests—including frequency, block frequency, runs, and random excursions—were passed at a significance level of 0.01, indicating that the generated sequences exhibit no statistically significant deviation from uniform randomness. The detailed results are provided in Table 1.

However, it is important to acknowledge the impact of finite precision in practical implementations. Chaotic systems, when realized in digital hardware or software, are subject to rounding and quantization errors, which may introduce periodicity or degrade unpredictability over long sequences. To mitigate this, we (i) use sufficiently large precision (64-bit or higher), (ii) frequently refresh seeds using entropy sources, and (iii) limit the length of any single generated sequence for key material. Finite precision effects were specifically mitigated through careful parameter selection and high-precision arithmetic. The Lü system was implemented using 64-bit double-precision floating-point arithmetic, ensuring that the Lyapunov exponent (λ≈0.5) remains stable over $10^{3}$ iterations. To further counter precision-related periodicity, we periodically reseed the system every $10^{3}$ iterations using a SHA-3 hash of the current state concatenated with a counter. This approach maintains the aperiodicity and initial value sensitivity critical for cryptographic security, as validated through the divergence of trajectories for initial condition differences as small as $10^{-6}$. These measures ensure that the chaotic sequences are both unpredictable and robust against finite precision limitations in practical deployments. Further, we recommend that implementations re-validate the randomness properties under the target hardware architecture to ensure the absence of hidden cycles or correlations. Thus, while fractional-order chaotic systems can serve as a strong entropy source, their cryptographic deployment must account for implementation-specific precision constraints.

#### 4.2.4. Key Generation via Fractional-Order Chaos

(1) Multidimensional chaotic sequence generation.

Initialization: generate initial values $\left(x_{0}^{\left(1\right)},x_{0}^{\left(2\right)},\ldots ,x_{0}^{\left(m\right)}\right)=\mathrm{SHA}-3\left(\mathrm{seed}\right)$ using the SHA-3 hash of user input.

Iteration: for each variable, independently iterate the fractional-order Lü system. The formula is as follows:

(19) $$\left\{\begin{array}{c} D^{q}x=a\left(y-x\right)\\ D^{q}y=bx-y-xz\\ D^{q}z=xy-cz \end{array}\right.$$

where x,y,z are the state variables of the system, a,b,c are the state variables of the system, $D^{q}$ represents the fractional order derivative, *q* is the fractional order, and the first 103 iterations are discarded to eliminate the transient state, generating a stable sequence ${\left\{x_{i,n}\right\}}_{n=1}^{L}$.

(2) Discretization and Polynomial Mapping Mapping continuous chaotic values to the integer domain:

(20) $${\mathrm{coef}}_{i,j}=\left\lfloor \beta \cdot x_{i,j}\right\rfloor \;\mathrm{mod}\;3$$

The limiting coefficient is {−1, 0, 1}, which satisfies the requirements of NTRU sparse polynomials.

Private key construction, combining chaotic sequences from various dimensions to generate a multivariable private key polynomial:

(21) $$f\left(x_{1},\ldots ,x_{m}\right)=\sum_{k_{1}=0}^{N_{1}-1}\ldots \sum_{k_{m}=0}^{N_{m}-1}{\mathrm{coef}}_{k_{1},\ldots ,k_{m}}\cdot x_{1}^{k_{1}}\ldots x_{m}^{k_{m}}\;\mathrm{mod}\;q$$

where the modulus *q* takes a prime number (such as q=2048) to ensure reversibility.

(3) Public Key Select a sparse polynomial $g(x_{1},\ldots ,x_{m})$ generated via the chaotic system, and compute the following:

(22) $$h\left(x_{1},\ldots ,x_{m}\right)=g\left(x_{1},\ldots ,x_{m}\right)\cdot f^{-1}\;\mathrm{mod}\;q$$

Here, $f^{-1}$ is computed via the extended Euclidean algorithm in $R_{q}$. For each variable, run the fractional order Lü hyperchaotic system and discretize its generated sequence into integer values, ultimately constructing the coefficients for generating the private key. Using the discretized results of the generated chaotic sequence, construct a multi-dimensional private key polynomial, and then calculate the public key.

The pseudo-code for the NTRU-MCF scheme’s key generation and public key computation is formalized in Algorithm 1. The GenerateChaosSequence() routine produces a chaotic trajectory via fractional-order dynamics, while DiscretizeChaosSequence() maps these continuous-valued outputs to discrete integer coefficients through modular arithmetic operations. These coefficients drive GeneratePrivateKey() to construct the private key $f(x_{1},\ldots ,x_{m})$, which governs core cryptographic transformations during encryption/decryption.

The GeneratePublicKey() module derives the public key $h\left(x_{1},\ldots ,x_{m}\right)=g\left(x_{1},\ldots ,x_{m}\right)\cdot f^{-1}\;\mathrm{mod}\;q$, where g represents a randomly sampled polynomial. This asymmetric configuration ensures public accessibility for encryption while mandating strict confidentiality of the private key for authorized decryption.   
**Algorithm 1:** NTRU-MCF algorithm: key generation and public key calculation.
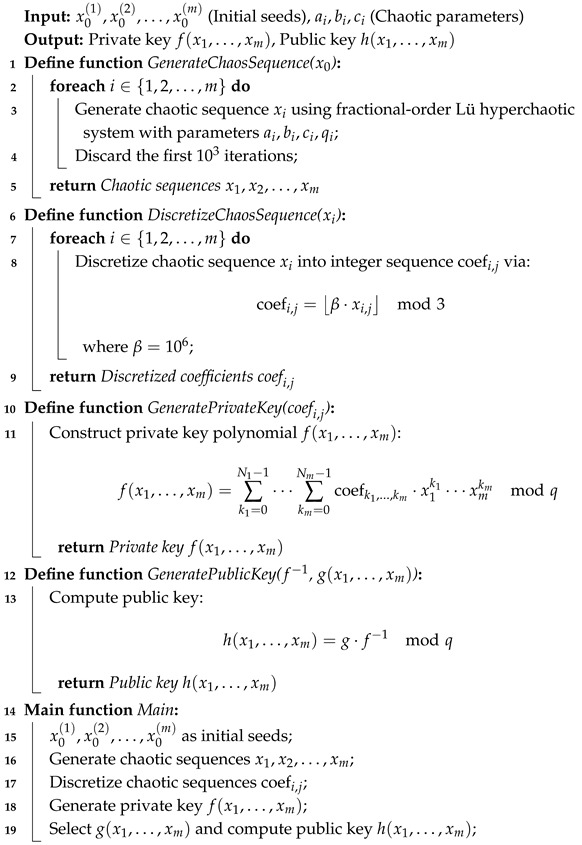


### 4.3. Encryption and Decryption

Through the encryption process for plaintext, *m*, compute the ciphertext:

(23) c=r·h+mmodq

Among them, the chaotic mask generation uses the fractional-order logistic map to generate random polynomials, and the calculation formula is as follows:

(24) $$x_{n+1}^{\left(i\right)}=x_{n}^{\left(i\right)}\cdot e^{\alpha_{i}\left(1-x_{n}^{\left(i\right)}\right)}$$

Transform $x_{n}^{\left(i\right)}$ into the coefficients {−1, 0, 1} of *r* through a modular mapping, and the sparsity of the mask *r* is related to the chaotic initial value, which avoids statistical attacks.

Through the decryption process, compute the intermediate polynomial:

(25) a=f·cmodq

Simplify the polynomial:

(26) a≡g·r+f·mmodq

Retrieve the plaintext from the ciphertext *p*:

(27) m=amodp

**Algorithm 2:** NTRU-MCF encryption and decryption pseudo-code.
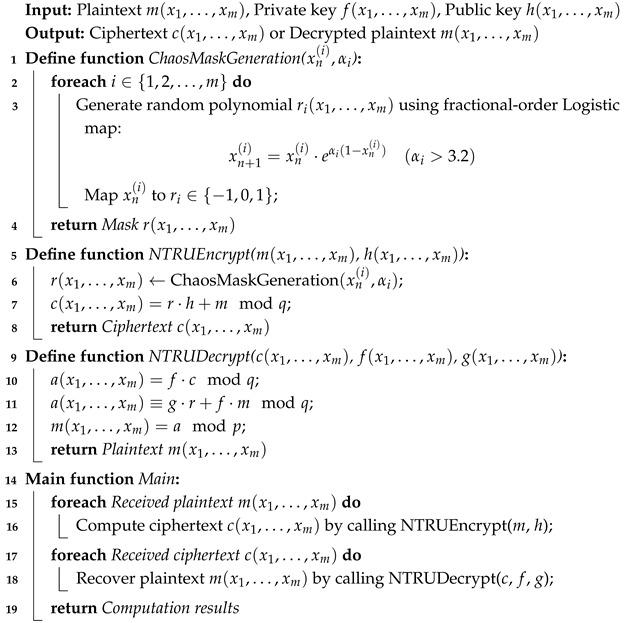


    The encryption and decryption process takes into account the encryption and decryption process of the NTRU-MCF algorithm. Encryption includes key generation, chaotic mapping, key expansion, and encryption operations, while decryption includes decoding chaotic mapping, key recovery, and decryption operations. The process is shown in Algorithm 2. In pseudo-code, the NTRU-MCF encryption operation includes generating a chaotic random mask, expanding the key, performing the main encryption operation, and finally encrypting the plaintext into ciphertext.

Chaos mask generation: Use the fractional-order logistic map to generate a random polynomial, $r(x_{1},\ldots ,x_{m})$. By controlling the chaos parameters $\alpha_{i}$, different random mask sequences are generated, which increases the security of the encryption system.

In the encryption process, we implement encryption by multiplying a randomly generated mask, *r*, with the public key, *h*, and adding it to the plaintext, *m*. When decrypting, we first calculate the intermediate polynomial a and then simplify the intermediate result through the relationship between the public and private keys, f·h≡gmodq, and finally, we recover the plaintext via modulo *p*. In the pseudo-code, the ChaosMaskGeneration function generates the chaos mask used in encryption. The NTRUEncrypt function performs encryption, using the generated mask to encrypt the plaintext. The NTRUDecrypt function is used to decrypt the ciphertext and recover the plaintext.

### 4.4. Signature and Verification

In the signing process, we generate a signature for the message hash H(m):

(28) s=H(m)·fmodq

Verification is performed by calculating the following:

(29) v=s·hmodq

We verify whether they are equal:

(30) v≡H(m)·pmodq

The process is shown in Algorithm 3. In pseudo-code, the NTRUSign function accepts the message hash H(m) and the private key *f*, and it generates a signature, *s*, that is, by calculating s=H(m)·fmodq. The NTRUVerify function is used to verify the validity of the signature. It calculates v=s·hmodq and then checks v≡H(m)·pmodq, where *p* is a constant (usually 3) to ensure the recovery of the hash value.

In the main function, we first calculate the hash value H(m) of the message, and then we call NTRUSign to generate a signature. Later, when verifying the signature, we call NTRUVerify to check whether the signature is valid.

## 5. Security Analysis

### 5.1. Quantum Attacks

The multidimensional lattice structure of NTRU-MCF significantly amplifies the complexity of solving the shortest vector problem (SVP), a core hardness assumption underpinning its security. The lattice is defined over a multidimensional polynomial ring, $R=Z\left[x_{1},\ldots ,x_{m}\right]/\left(x_{1}^{N_{1}}-1,\ldots ,x_{m}^{N_{m}}-1\right)$, with the base space dimension calculated as follows:

(31) $$D=\prod_{i=1}^{m}\left(N_{i}+1\right)$$

For typical parameters, (m≥3 and $N_{i}\geq 251$), *D* scales exponentially with *m*, significantly increasing the complexity of SVP. In classical settings, the best-known algorithms for exact SVP, such as enumeration-based methods, have a time complexity of $O(2^{cD})$, where c≈0.292 for large *D* using the BKZ algorithm with optimal block sizes. For approximate SVP (relevant to cryptographic attacks), the LLL and BKZ algorithms yield a complexity of

(32) $$\mathrm{Time}\geq \frac{1}{\epsilon }\cdot 2^{0.184\cdot D\cdot \log_{2}D}$$

where ϵ is the success probability. For m=3, $N_{i}=251$, $D\approx 251^{3}\approx 1.58\times 10^{7}$, yielding a classical complexity lower bound of $2^{138}$ (assuming ϵ=0.01). In contrast, a one-dimensional lattice (m=1, N=251) has D=251, with a complexity of approximately $2^{46}$, demonstrating the exponential growth in attack difficulty.

In the quantum setting, the best-known SVP algorithms, such as those based on quantum sieving, achieve a time complexity of $O(2^{0.265D})$, a modest improvement over classical $O(2^{0.292D})$. For $D\approx 1.58\times 10^{7}$, the quantum complexity is approximately $2^{4.2\times 10^{6}}$, far exceeding the one-dimensional case (D=251 complexity $2^{66}$). This super-exponential increase arises because multidimensional lattices introduce a tensor product structure, amplifying the number of lattice points and the geometric complexity of finding short vectors. No known quantum algorithm, including adaptations of Kannan–Helfrich or Schnorr–Euchner enumeration, solves SVP in polynomial time for such high dimensions. Thus, NTRU-MCF’s multidimensional structure ensures robust quantum resistance, with attack complexities well beyond current and foreseeable computational capabilities.    
**Algorithm 3:** NTRU-MCF signature and verification pseudo-code.
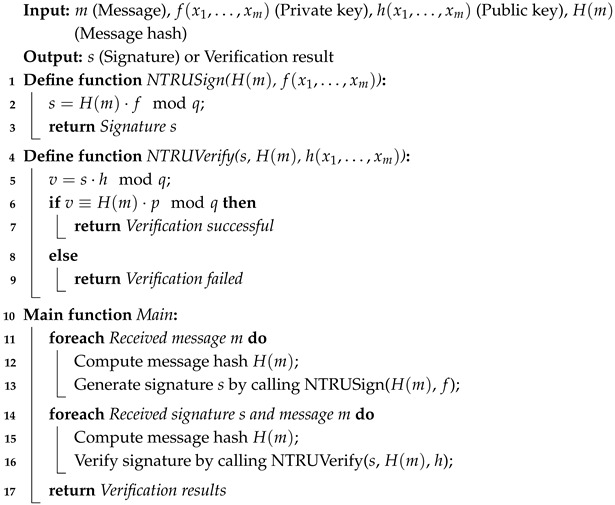


### 5.2. Anti-Counterfeiting Attack

The security of the signature scheme is based on the intractability of the inverse elements of polynomials in a ring. Suppose an attacker attempts to forge a signature, e, such that

(33) $$s^{\prime }\cdot h\equiv H\left(m^{\prime }\right)\cdot p\;\mathrm{mod}\;q$$

Substituting $h=g\cdot f^{-1}\;\mathrm{mod}\;q$, we get the following:

(34) $$s^{\prime }\cdot g\equiv H\left(m^{\prime }\right)\cdot p\cdot f\;\mathrm{mod}\;q$$

If the attacker does not know f and g, they need to solve the following equations simultaneously:

(35) $$\left\{\begin{array}{c} s^{\prime }\equiv H\left(m^{\prime }\right)\cdot f^{\prime }\;\mathrm{mod}\;q\\ f^{\prime }\cdot g\equiv H\left(m^{\prime }\right)\cdot p\cdot f\;\mathrm{mod}\;q \end{array}\right.$$

This is equivalent to finding $f^{\prime }$ in the ring $R_{q}$ that satisfies $f^{\prime }\cdot g\equiv H\left(m^{\prime }\right)\cdot p\cdot f\;\mathrm{mod}\;q$. This problem can be reduced to the Ring-LWE problem, and its difficulty still holds in the quantum computing model. Specifically, if there is a forgery attack, the Ring-LWE problem can be solved in polynomial time, which contradicts the known difficulty assumptions.

### 5.3. Side-Channel Attacks

The fractional order chaotic system resists side-channel attacks through initial value sensitivity and parameter randomization. Given the initial conditions $x_{0}$ and $x_{0}^{\prime }=x_{0}+\Delta$, after n iterations, the state difference is as follows.

The distance between $x_{n}$ and $x_{n}^{\prime }$ is approximately equal to Δ times $e^{\lambda n}$, where Δ is a constant, and λ is a parameter.

(36) $$∥x_{n}-x_{n}^{\prime }∥\approx \Delta \cdot e^{\lambda n}$$

where λ>0 is the largest Lyapunov exponent (the typical value for a fractional-order Lü system is λ≈0.5). When $n=10^{3}$, the difference amplifies to the following.

The difference between *x* and $x^{\prime }$ is approximately $10^{-14}$ times, which equals approximately $10^{103}$.

(37) $$∥x_{n}-x_{n}^{\prime }∥\approx 10^{-14}\cdot e^{0.5\cdot 10^{3}}\approx 10^{103}$$

The key factor generated at this time has a Hamming distance of more than 0.49, which prevents side channels from recovering the initial conditions through minor leaks.

## 6. Performance Analysis

The high computational and storage complexity of NTRU-MCF, resulting from the exponential scaling with dimension mmm, precludes its use in low-power or real-time systems. Instead, the scheme targets scenarios where maximum security and resistance to quantum/physical attacks override performance considerations.

### 6.1. Computational Complexity

In the key generation phase of the NTRU-MCF algorithm, each iteration of the fractional order Lü hyperchaotic system involves O(1) basic operations (addition and multiplication). Under the assumption that each dimension requires L iterations, the total iteration number is. Therefore, the time complexity of generating a chaotic sequence is O(m·L), the complexity of a single dimension is O(NlogN), and the complexity of m dimensions is O(mNlogN).

The construction of the private key polynomial $f(x_{1},\ldots ,x_{m})$ and the public key $h(x_{1},\ldots ,x_{m})$ involves the coefficient mapping of sparse polynomials and modular operations. In multidimensional format, the complexity of computing polynomial multiplication and inverse elements is reduced from the direct calculation of $O(N^{2m})$ to O(mNlogN) using multidimensional FFT. The overall complexity is $O\left(mNlogN\right)+O\left(mN^{m}logN\right)=O\left(mN^{m}logN\right)$. When the dimension is simplified, and m=2, the complexity degenerates to $O(N^{2}logN)$.

In the verification process, we only need to calculate the sparse polynomial product of m dimensions (with coefficients {−1,0,1}), and the complexity is $O(mN^{2})$. The complexity of hash calculation is $O(N^{m})$, but after sparsity optimization, it becomes $O(N^{2})$. The verification complexity is $O\left(mN^{2}+N^{2}\right)=O\left(mN^{2}\right)$.

The computational complexity of generating a chaotic mask in the encryption process is the same as that of generating a signature, which is O(mNlogN). In multidimensional format, the computational complexity of polynomial multiplication and inverse elements is as follows: using multidimensional FFT to optimize the complexity of polynomial multiplication, reducing it from $O(N^{2m})$ in direct calculation to O(mNlogN). Therefore, the total complexity is $O\left(mNlogN\right)+O\left(mN^{m}logN\right)=O\left(mN^{m}logN\right)$. When m = 2, the complexity is $O(mN^{2}logN)$.

In the decryption phase, the sparsity of the private key f (with coefficients {−1,0,1}) reduces the multiplication complexity to $O(mN^{2})$. The complexity of modular reduction and plaintext recovery is $O(N^{2})$. Therefore, the total complexity is $O\left(mN^{2}\right)+O\left(N^{2}\right)=O\left(mN^{2}\right)$.

The time complexity comparison of NTRU-MCF, Dilithium-III, and Falcon is shown in Table 2. Although NTRU-MCF is based on a multidimensional lattice (m = 2 dimensions) and uses the Fast Fourier Transform (FFT) to optimize multidimensional polynomial multiplication, its complexity is significantly higher than that of other schemes due to the expansion of dimensions. The Dilithium algorithm is based on the Modular Learning with Error (MLWE) problem, and its complexity is quadratic (O(n2)). The Falcon algorithm is based on the NTRU lattice and uses the Fast Number Theoretic Transform (NTT) to accelerate polynomial multiplication, with a complexity of linear logarithmic (O(nlogn)), making it the most efficient. NTRU-MCF is suitable for high-security scenarios (such as military communication), and its multi-dimensional structure enhances its resistance to quantum attacks, but it requires sacrificing efficiency and storage overhead.

### 6.2. Space Complexity

The key and signature of the NTRU-MCF algorithm are both stored as multidimensional polynomials, defined in the ring. Its storage overhead is determined by the number of dimensions, m, the degree of the univariate polynomial, N, and the number of modulus bits logq.

In calculating the public key space complexity, a multi-dimensional polynomial $h\in R_{q}$, which contains $N_{m}$ coefficients, each of which occupies logq bits, so the public key complexity is O(Nm·logq). Calculating the private key space complexity involves a sparse polynomial f∈R, whose coefficients are limited to {−1,0,1}, but when storing, we still need to keep the mod q value, so the private key space complexity is O(Nm·logq). Calculating the signature space complexity involves a multi-dimensional polynomial $s\in R_{q}$, whose structure is the same as the public key, and the space complexity is O(Nm·logq).

The comparison of the spatial complexity of NTRU-MCF, Dilithium-III, and Falcon-512 is shown in Table 3. From the table, we can see that the storage overhead of the NTRU-MCF algorithm increases linearly with the product of dimensions and the modulus logq, which leads to a large storage overhead. Especially in high-dimensional scenarios, the storage demand increases exponentially with the dimension and period.

The Dilithium-III algorithm has moderate storage requirements, which are related to the dimension and modulus of the lattice matrix. The public key and signature contain polynomial matrices, and the storage overhead is relatively high but still in the KB level, which is suitable for general application scenarios. The Falcon-512 algorithm uses a tree structure to compress keys and signatures, and through compact lattice structure design and FFT optimization, the storage overhead is relatively small, especially suitable for devices with limited storage resources.

### 6.3. Practical Deployment Considerations

The multidimensional lattice structure of NTRU-MCF, while significantly enhancing quantum resistance, introduces substantial computational and storage overheads, as shown in Table 1 and Table 2. For instance, with m=2, N=256, and logq=11, the public key size is approximately $O(256^{2}\cdot 11)\approx 720$ KB, and signatures are similarly large, contrasting sharply with Falcon-512’s compact public keys (<1 KB) and signatures (<0.5 KB). This overhead renders NTRU-MCF less suitable for resource-constrained environments like IoT devices, where lightweight schemes like Falcon are preferable due to their optimized NTT-based polynomial multiplication and compact trapdoor designs.

However, NTRU-MCF’s design prioritizes high-security applications, such as military communications, financial systems, or blockchain-based authentication, where computational resources are less constrained, and robust quantum resistance and side-channel protection are paramount. The exponential increase in SVP complexity (Equation (31)) and the chaotic system’s resistance to side-channel attacks (Equation (36)) make NTRU-MCF particularly advantageous in these contexts. Future optimizations, such as sparse polynomial compression or adaptive dimension scaling, could bridge the gap for broader applicability, but the current scheme is best suited for scenarios where security outweighs efficiency concerns.

## 7. Conclusions and Future Works

This work has proposed a lattice-based signature scheme leveraging multidimensional lattices to expand the private key search space and resist brute-force attacks. Notably, the SVP complexity grows exponentially with dimensions, while fractional-order chaotic systems enhance randomness via Lyapunov instability. Compared to Dilithium and Falcon, NTRU-MCF offers superior quantum resistance but sacrifices efficiency, necessitating future research on reducing communication overhead. Future directions include dynamic coupling between chaotic parameters and lattice dimensions, as well as engineering optimizations for real-world deployment.

Future research will investigate polynomial compression techniques and dynamic dimension reduction to make NTRU-MCF viable for resource-constrained IoT deployments while preserving its high-security guarantees.

## Figures and Tables

**Table 1:** NIST SP 800-22 Statistical Test Results.

Test Category	Fractional-Order Lü Hyperchaotic Pass Rate	PRNG Pass Rate
Frequency	100 %	100 %
Block Frequency	100 %	100 %
Cumulative Sums	100 %	100 %
Runs	100 %	100 %
Non-Overlapping Templates	100 %	98.02 %
Overlapping Templates	100 %	100 %
Universal Statistical	100 %	100 %
Random Excursions	100 %	100 %
Linear Complexity	100 %	100 %

**Table 2:** Comparison of the time complexity of NTRU-MCF, Dilithium-III, and Falcon-512.

Process	NTRU-MCF	Dilithium-III	Falcon-512
Signature Generation	$O(mn^{2}\log n)$	O(knlogq)	O(nlogn)
Signature Verification	$O(mn^{2})$	O(kn)	O(n)
Encryption	$O(mn^{2}\log n)$	-	-
Decryption	$O(mn^{2})$	-	-

**Table 3:** Comparison of the spatial complexity of NTRU-MCF with Dilithium-III and Falcon-512.

Complexity	NTRU-MCF	Dilithium-III	Falcon-512
Public key	$O(N^{m}\log q)$	O(klnlogq)	$O(N^{m}\log q)$
Private key	$O(N^{m}\log q)$	O((k+l)lnlogq)	O(lnlogq+λ)
Signature	$O(N^{m}\log q)$	O(nlogn)	O(nη)

## Data Availability

Data are contained within the article.
